# Favorable therapeutic response after anti-Mesothelin antibody–drug conjugate treatment requires high expression of Mesothelin in tumor cells

**DOI:** 10.1007/s00404-020-05734-9

**Published:** 2020-08-19

**Authors:** Lea Lazzerini, Korinna Jöhrens, Jalid Sehouli, Günter Cichon

**Affiliations:** 1 Department of Gynecology, Charité, Universitätsmedizin Berlin, Campus Benjamin Franklin, Hindenburgdamm 30, 12200 Berlin, Germany; 2 Department of Pathology, Universitätsklinikum Carl Gustav Carus Dresden, Schubertstrasse 15, 01307 Dresden, Germany

**Keywords:** Mesothelin, Cancer, Anti-mesothelin drug conjugates, Anetumab ravtansine

## Abstract

**Purpose:**

The cell surface glycoprotein Mesothelin is overexpressed in ovarian, fallopian tube, endometrial, cervical and primary peritoneal cancer and, therefore, might become a particular interesting tumor target in gynecologic oncology. However, even in malignant tumors of the same entity the level of Mesothelin expression varies between individuals, hence it can be expected that the response to Mesothelin-targeting therapies will be variable as well. In this study we explored the therapeutic potency of a novel anti-Mesothelin antibody–drug conjugate (Anetumab ravtansine) as a function of Mesothelin expression in the targeted tumor cells.

**Methods:**

Anti-tumor activity studies were performed in human uterine xenograft tumor models that express Mesothelin at high, moderate or low levels. The antibody–drug conjugate (ADC) was applied in varying doses ranging from 2 to 15 mg/kg at variable intervals in tumor bearing SCID/beige mice and the impact on tumor growth was monitored.

**Results:**

The therapeutic response to the anti-Mesothelin ADC correlated closely with the level of Mesothelin expression in tumor cells. Within the applied dose levels complete tumor regression was achieved only in tumors which expressed Mesothelin at particularly high levels (Hela cell tumors). The application of high anti-Mesothelin ADC doses less frequently was more efficious than giving lower doses at a higher frequency.

**Conclusion:**

The studies confirm the great therapeutic potential of Anetumab ravtansine. However, a favorable treatment outcome requires strong Mesothelin expression in tumor cells. Future clinical trials may benefit from a more rigorous selection of appropriate patients based on the level of Mesothelin expression in their tumor tissue. If, in addition, it is possible to better control side effects by introducing protective measures and by doing so to increase the maximum tolerated dose, Anetumab ravtansine has the potency to become a valuable therapeutic tool, especially in the field of gynecological oncology.

## Introduction

Mesothelin was first described by Chang and Pastan 1992 as a 40 kDa membrane glycoprotein which is predominantly expressed in mesodermal tissue [1, 2]. Mesothelin and its binding partner MUC16 (CA125) play a role in cell adhesion, whereas other physiologic functions are not known so far [3]. Mesothelin knock out mice show a normal phenotype indicating that this glycoprotein does not play an essential role in normal cellular physiology [4].

Mesothelin as a tumor target is particularly interesting in gynecology since the female inner genital organs are formed by the middle germ layer, the mesoderm. The upper part of the vagina, the uterus and the fallopian tubes develop from the mesodermal Müllerian duct (paramesonephric duct) [5]. Under physiological condition Mesothelin is expressed in these tissues, however the expression level increases substantially during malignant transformation [6]. Particularly high Mesothelin expression levels are found in the majority of pancreatic cancers, in ovarian cancer, in endometrium cancer and in cervical adenocarcinomas [7, 8]. Whether an increased Mesothelin expression provides a growth advantage for the tumor or has an impact on the prognosis is discussed controversially. Some reports describe high Mesothelin expression to be associated with a more aggressive behavior of the tumor and worse prognosis in breast, lung and gastric cancer [9–11] while others found no impact of Mesothelin overexpression on prognosis in gastric and breast cancer, or even an improved prognosis, respectively [12, 13].

The fact that membrane bound Mesothelin levels are increased in several human cancers and the protein is accessible from the extracellular space makes it a target for novel Mesothelin directed antibody-based therapies.

In the present study we explored the therapeutic effect of the antibody–drug conjugate Anetumab ravtansine. Anetumab ravtansine is an ADC in which a monoclonal antibody specific for human Mesothelin is bound to a highly toxic maytansinoid (DM4) [14]. Maytansoids are natural occurring agents which are isolated from plants and shrubs growing in Africa and other warm areas [15]. As a conjugate to an anti-Mesothelin antibody Anetumab ravtansine bounds to Mesothelin and becomes internalized by Mesothelin-positive tumor cells. Following internalization degrading enzymes release the cytotoxic maytansinoid DM4 which then acts as a microtubule destabilizer and induces cell cycle arrest and apoptosis [16].

Preclinical results and early clinical studies employing Anetumab ravtansine were very promising [14, 16]. In a recent phase II trial the therapeutic potential of Anetumab ravtansine was explored as second line therapy in 248 mesothelioma patients who were no longer responsive to the standard therapy (Cisplatin/Pemetrexed). Unfortunately, in this trial the progression free survival of treated patients was not improved by the treatment compared to patients who received the standard therapy with Vinorelbine only [17]. Despite this drawback Mesothelin holds a potential as a novel target in oncology but it might be neccessary to revise the application regime, perform a stronger selection of patients suitable for treatment and improve associated measures which help to better control side effects and which might allow to increase the applied dose.

To explore the therapeutic potency and to optimize application regimes we performed dose-efficiency studies in three uterus derived human tumor models which express Mesothelin at variable levels.

## Material and methods

### Generation of xenotransplant tumors in Scid beige mice

#### Animal experiments

Animal experiments were approved by the state office for health and social affairs Berlin (Landesamt für Gesundheit und Soziales (Berlin); reference number G 0262/10. SCID/beige mice were purchased from Charles River laboratories (Sulzfeld, Germany). Hela and Caski cells were obtained from the DSMZ (Deutsche Sammlung für Mikrorganismen und Zellkulturen, Braunschweig, Germany) and the Cx-03 cell line was established in our lab from a human uterine carcino sarcoma of the uterus [18]. Cells were cultured under standard conditions (DMEM, 10% FCS, Pen/Strep; Thermo Fisher Scientific, Darmstadt, Germany). The anti-Mesothelin ADC Anetumab ravtansine was provided by Bayer AG (Berlin, Germany).

For generation of xenotransplant tumors 1 × 10e–5 trypsinized and washed vital tumor cells in 100 µl DMEM (without Pen/Strep and FBS) were injected subcutaneously into the back of the mice (27 gauge needle). Outgrowth of Hela and Cx-03 tumors took about 5–6 weeks and of Caski tumors about 7–8 weeks.

The treatment with the anti-Mesothelin ADC was started when tumors had reached a size of 5–10 mm diameter. Animals in the treatment groups (*n* = 4–7) received 2 mg/kg, 5 mg/kg, 10 mg/kg or 15 mg/kg Anetumab ravtansine in 200 µl injection buffer (PBS) in different intervals (weekly, twice weekly or every 3 weeks) by i.p. injection. Animals in the control group received injection buffer only. Tumor size was monitored by caliper.

### Mesothelin staining of xenotransplant tumors

Formalin fixed paraffin embedded tissue (FFPE) of Hela, Cx-03 and Caski tumors were stained with haematoxylin and eosin (HE) for histological tumor evaluation. To asses individual differences in expression patterns, five different xenograft tumors derived from each cell line were analyzed for Mesothelin expression. Mesothelin expression was determined on paraffin tissue sections by IHC staining using a Leica Bond™ polymer fully refine detection system. The employed anti-Mesothelin antibody was purchased from Thermo scientific (clone 5B2, 1: 100, Thermo Scientific MS-1320). A mesothelioma cell line was employed as positive control.

For determination of the Mesothelin expression level three representative high power fields from tumor samples were selected and the mean H-score was calculated as the product of the relative proportion of Mesothelin-positive tumor cells (percent) and IHC staining intensity (1 + , 2 + 3 +) as follows: 1 × (% cells 1 +) + 2 × (% cells 2 +) + 3 × (% cells 3 +). The highest possible score is 300.

## Results

### Mesothelin expression levels in three uterine xenotransplant tumors

**Fig. 1:**
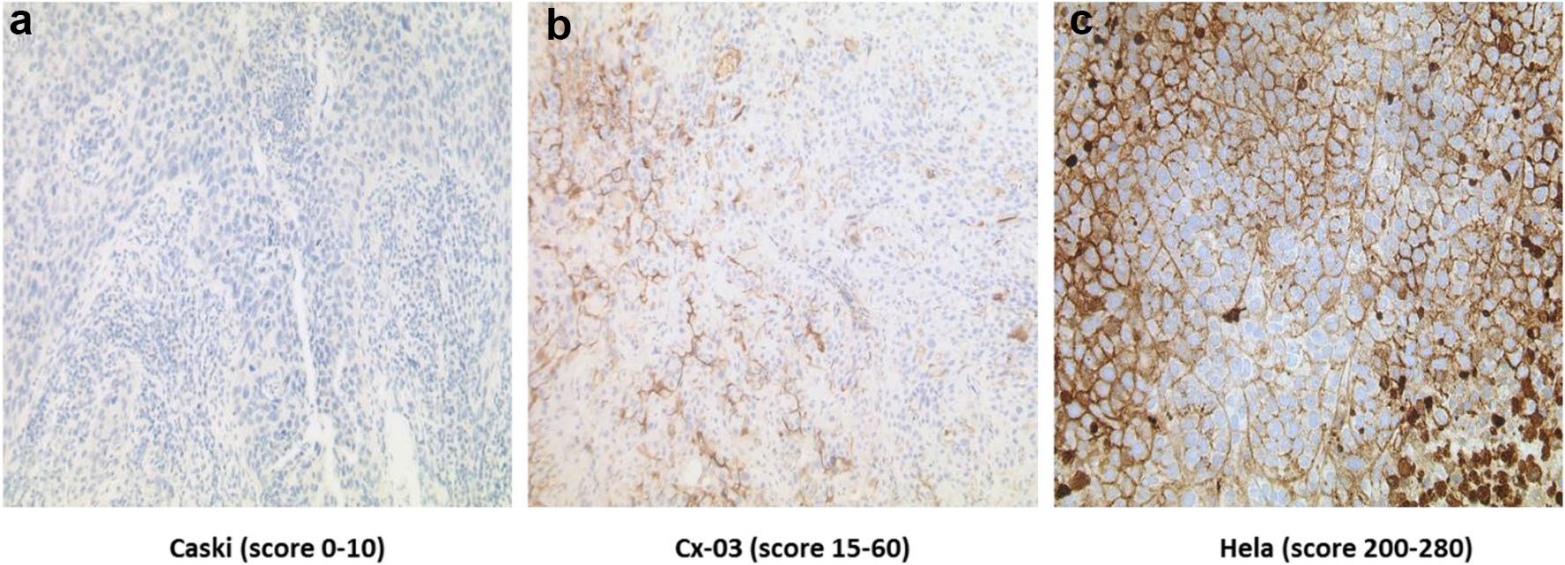
Mesothelin expression patterns in three different uterine carcinoma models. Caski (**a**) and HeLa (**c**) tumors are derived from cervical squamous cell carcinoma lines and Cx-03 tumors (**b**) from a uterine carcinosarcoma. In Caski tumors Mesothelin expression is mostly absent while Cx-03 tumors show moderate and Hela tumors high Mesothelin expression levels

To determine the average Mesothelin expression levels in the selected xenograft models, five xenograft tumors derived from each cell line were examined. In Caski cell xenotransplant tumors (Fig. 1a) Mesothelin expression was barely detectable (score 0–10), Cx-03 tumors (Fig. 1b) showed moderate expression levels (score 15–60) and Hela cell derived tumors (Fig. 1c) expressed Mesothelin to high levels (score 200–280).

### Therapeutic response to anti-Mesothelin ADC Anetumab ravtansine

To evaluate the therapeutic potential of an anti-Mesothelin ADC dose-efficiency studies were started when tumors had reached a size of 5–10 mm. In a first experiment animals received increasing doses from 2 mg/kg up 10 mg/kg twice weekly by intraperitoneal injection.

**Fig. 2:**
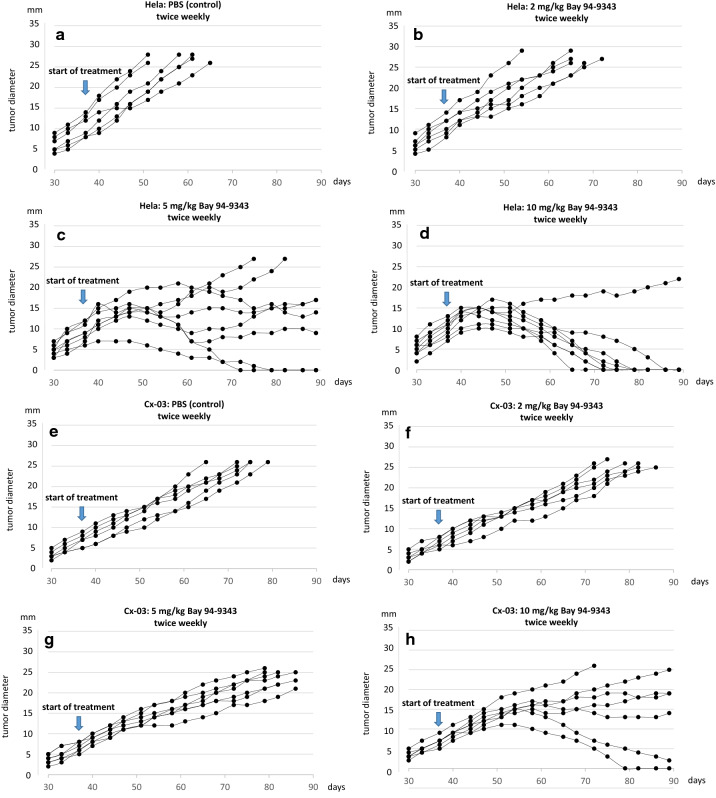

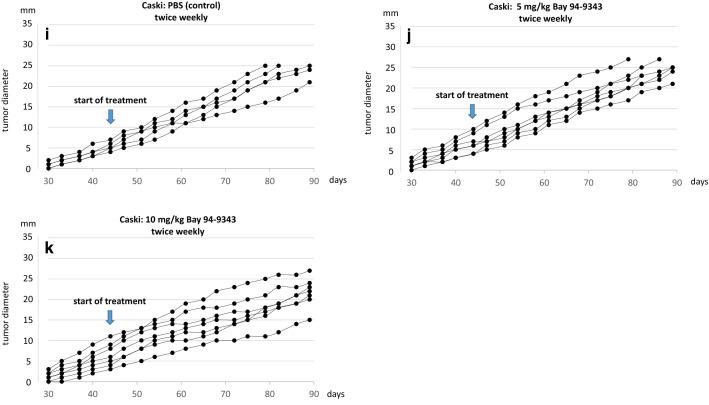
Among the three explored uterine tumor models Hela cell derived tumors (**a**–**d**) show the highest sensitivity to the treatment with the anti-Mesothelin ADC. After applying 10 mg/kg twice weekly complete tumor remission is observed in 6 of 7 animals (**d**). Compared to Hela tumors Cx-03 tumors (**e**–**h**) show a reduced response, but a significant growth retardation is observed after applying a dose 10 mg/kg twice weekly and complete response in 1 animal (**h**). Caski cell tumor (**i**–**k**) are mostly unresponsive to the treatment and only a mild reduction in growth is observed at the highest ADC dose (**k**)

The results demonstrate a positive correlation between the level of Mesothelin expression in tumor cells and the sensitivity to the anti-Mesothelin ADC (Fig. 2). In Hela cell tumors a dose of 2 mg/kg applied twice weekly induced a mild retardation in growth speed but did not prevent tumor progression (Fig. 2b). Increasing the dose to 5 mg/kg twice weekly induced a sustained growth control in 4 of 8 animals and complete tumor remission in 2 of 8 animals (Fig. 2c). A further dose increase to 10 mg/kg twice weekly led to complete tumor remission in 6 of 7 animals (Fig. 2d).

Compared to Hela cell derived xenograft tumors Cx-03 tumors showed a reduced sensitivity against the MSLN-ADC. Applying a dose 2 mg/kg of the ADC twice weekly had no impact on growth speed compared to the control group (Fig. 2e, f). Increasing the dose to 5 mg/kg twice weekly induced a retardation in growth but did not prevent outgrowth of tumors (Fig. 2g). At a dose of 10 mg/kg 4 of 7 animals showed a sustained growth control while in one animal tumor disappeared completely (Fig. 2h).

In Caski cell tumors ADC treatment remained mostly uneffective (Fig. 2i–h). Only in the highest dose group (10 mg/kg twice weekly) a mild retardation in growth was noted which did not prevent tumor progression (Fig. 2i–k).

The first measurable response to the treatment usually occurred with a delay of 10–14 days. Even in the highest dose groups (10 mg ADC/kg) it took 3–4 applications to achieve a visible growth arrest (e.g. Fig. 2d).

**Fig. 3:**
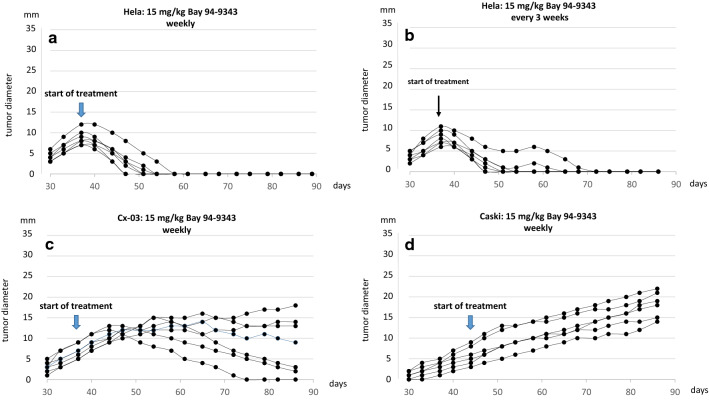
Applying a dose of 15 mg/kg weekly (**a**) or every three weeks (**b**) leads to complete tumor regression in Hela cell tumors. In Cx-03 (**c**) and Caski cell tumors (**d**) no substantial difference was observed compared to the previous protocol (10 mg/kg twice weekly)

To explore the impact of further dose increase 15 mg/kg of the MSLN-ADC were applied at longer intervals (weekly and every 3 weeks) (Fig. 3). In this setting the therapeutic response occurred more rapidly and a complete tumor reduction was achieved in 6 of 6 Hela tumor bearing mice within 3 weeks (Fig. 3a). Giving the MSLN-ADC in longer intervals (once every 3 weeks) still provided a remission rate of 100% with some minor delay (Fig. 3b). In Cx-03 the therapeutic response was less pronounced, but MSLN-ADC still induced complete tumor remission in 1 of 7, partial tumor remission in 2 of 7 and growth arrest in the other 4 animals (Fig. 3c). In the Caski model only a minor reduction in growth was noticed.

**Fig. 4:**
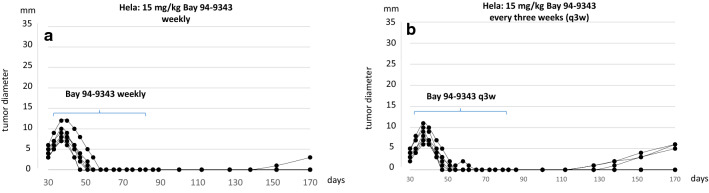
70 days after termination of the treatment tumor recurrence occurred in 1 of 6 animals in the group which had received weekly ADC applications and three recurrences were noticed after applying the ADC every 3 weeks

In the Hela group the repetitive MSLN-ADC application was stopped at day 90 after tumor cell inoculation and animals were further observed for additional 80 days. Tumor recurrence occured in only 1 of 6 animals of the 15 mg/kg weekly group (Fig. 4a) and 3 of 6 animals who received the ADC every 3 weeks (Fig. 4b).

## Discussion

In the present study three human uterine tumor models which express Mesothelin at different levels were used to explore the efficacy of an anti-Mesothelin antibody drug conjugate (Anetumab ravtansine). Hela and Caski cells were originally derived from human squamous cell carcinoma of the cervix and Cx-03 from a human uterine carcinosarcoma. Hela cell xenograft tumors express Mesothelin at high levels, low Mesothelin expression is found in Cx-03 cell tumors and no Mesothelin expression is detected in Caski xenograft tumors. In this study MSLN-ADC application was initially performed in short intervals (twice weekly in doses ranging from 2 to 10 mg/kg). The antibody drug conjugates were administered intraperitoneally, which ensures more reliable dosing, particularly in the case of repeated applications. After intraperitoneal administration, antibodies and antibody drug conjugate are well absorbed and lead to intratumoral drug concentrations close to that of intravenous administrations [19–21].

As expected, the therapeutic response was dependent on the applied dose and the expression level of Mesothelin in the xenograft tumors (Figs. 1, 2). In Hela derived tumors a dose of 10 mg/kg twice weekly induced complete tumor remission in more than 80% of animals, while in Cx-03 tumors which expressed Mesothelin at 20–30% compared to Hela tumors substantial tumor remission was noticed in 2 animals only and stable disease in the rest of the cohorte. The difference in the response rate of Hela and Cx-03 tumors corresponded quite well to the level of their Mesothelin expression. Caski cell xenografts who did not express Mesothelin, remained mostly unresponsive to MSLN-ADC treatment.

Looking at the tumor growth curves it was recognized that a reduction of tumor mass did not occur immediately after applying the first dose of ADC but rather took 3–4 applications within at least 7–14 days. When applying corresponding ADC doses to cell culture viability is decreasing within 24 h (data not shown). The different behavior of the tumor tissue in vivo may be due to deviating pharmacokinetic conditions and different tumor penetration patterns of ADC.

The half life of Anetumab Ravatasine in mice (in 20 mg/kg) is about 80 h [22–24]. Hence, it can be expected that applying ADC twice weekly some accumulation might take place. We conclude from our experiments that a relevant reduction of tumor mass might only occur after reaching a sufficient intratumoral threshold concentration and that it is a high peak concentration rather than a high accumulative dose over time that provides a sufficient therapeutic response.

Efficient transvascular penetration and internalization of ADCs into tumor tissue is a prerequisite for a therapeutic effect. However, ADC distribution in tumor tissues is known to be nonuniform. It depends on the molecular size of the ADC and its affinity to the target antigen, the vascularization and capillary permeabilty of the tumor, intratumoral antigen distribution, intratumoral interstitial pressure and the grade of necrosis. John Weinstein and his colleagues provided evidence for another phenomenon which they called a ‘binding site barrier’ [25–27]. After initial intravenous application the first wave of the ADC penetrates the tumor tissues by trafficking through the intratumoral capillary walls and forms a dense layer of ADC-antigen complexes around the capillary which hinders the deep and homogenous penetration of subsequently inflowing ADCs. They have shown that the barrier could be in part overcome by a dose increase, however, as a consequence of this phenomenon serum levels and intratumoral ADC concentration might not behave in a linear mode. As long as the intratumoral ADC levels are not sufficient the therapeutic response will remain unsatisfactory. For this reason the application of high doses of ADC in longer intervals might provide a better outcome compared to the more frequent application of lower doses. This conclusion is in good agreement with the outcome of the second series of experiments. Applying a dose of 15 mg/kg weekly or every 3 weeks provides a better outcome than giving 10 mg/kg twice weekly (Fig. 3).

In humans physiological expression of Mesothelin is found in epithelial layers of the pleura, the peritoneum and the pericard, in salivary glands, in the bone marrow, the cornea and in intestinal tissues [28]. Utilizing Mesothelin as a tumor target is therefore afflicted with unwanted cross-reactivity. Keratitis, neuropathy, fatigue, anorexia, asthenia, diarrhea and LFT increase are the most common drug-related laboratory abnormalities which restrict the maximum tolerated dose in clinical trials to 6.5 mg/kg (q3w) [29].

In mouse models MSLN-ADC can be administered in significantly higher doses since Anetumab ravtansine does not crossreact with murine Mesothelin. Considering the different pharmacokinetics between humans and mice a dose of 6.5 mg/kg q3w (MTD in humans) corresponds to 15 mg/kg q2w in mice [14].

In a second series of experiments it was shown that it a high peak level of the ADC rather than a high accumulative dose which provides and improved outcome (Fig. 3). This study confirms the potential of Anetumab ravtansine as an anti-tumor agent, however, the results suggest that aiming on a convincing clinical outcome a further dose increase and a restriction to patients displaying a strong intratumoral Mesothelin expression might be necessary. The screening and selection of patients suitable for Mesothelin-targeting therapies on the basis of their Mesothelin expression in tumor biopsies is easy to perform and daily routine in other tumor entities (e.g. HER2/neu in breast cancer). The major challenge seems to be the further increase of the applied dose in conjugation with a better control of side effects.

Mesothelin is a particular relevant and promising target especially in gynecologic oncology but it might be necessary to step back to suitable preclinical models to explore the benefit of antinflammatory and protective measures which help to control side effects and which might allow to increase the maximum tolerated doses.
